# Scientometric Data and Open Access Publication Policies of Clinical Allergy and Immunology Journals

**DOI:** 10.7759/cureus.13564

**Published:** 2021-02-26

**Authors:** Gokhan Tazegul, Emre Emre

**Affiliations:** 1 Internal Medicine, Ankara Polatlı Duatepe State Hospital, Ankara, TUR; 2 Immunology and Allergy, Hatay State Hospital, Antakya, TUR

**Keywords:** scientometrics, medical research, open access publishing, hypersensitivity, allergy and immunology

## Abstract

Introduction

The scientific merit of a paper and its ability to reach broader audiences is essential for scientific impact. Thus, scientific merit measurements are made by scientometric indexes, and journals are increasingly using published papers as open access (OA). In this study, we present the scientometric data for journals published in clinical allergy and immunology and compare the scientometric data of journals in terms of their all-OA and hybrid-OA publication policies.

Methods

Data were obtained from Clarivate Analytics InCites, Scimago Journal & Country Rank, and journal websites. A total of 35 journals were evaluated for bibliometric data, journal impact factor (JIF), scientific journal ranking (SJR), Eigenfactor score (ES), and Hirsch index (h-index). US dollars (USD) were used for the requested article publishing charge (APC).

Results

The most common publication policy was hybrid-OA (n = 20). The median OA publishing APC was 3000 USD. Hybrid-OA journals charged a higher APC than all-OA journals (3570 USD vs. 675 USD, p = 0.0001). Very strong positive correlations were observed between SJR and JIF and between ES and h-index. All the journals in the h-index and ES first quartiles were hybrid-OA journals.

Conclusion

Based on these results, we recommend the use of SJR and ES together to evaluate journals in clinical allergy and immunology. Although there is a wide APC gap between all-OA and hybrid-OA journals, all journals within the first quartiles for h-index and ES were hybrid-OA. Our results conflict with the literature stating that the OA publication model's usage causes an increase in citation counts.

## Introduction

In the ever-growing field of scientific publishing, it is necessary to compare and rank the quality of individuals, institutions, and journals. These data are used for various purposes, such as selecting which journals to subscribe to and submitting articles to, calculating the effect of publications, and deciding on academic promotions and grants. Such measurements of scientific merit are typically made by scientometric indexes [1].

All scientometric indexes consider a paper's citation count; however, the articles and citations taken into account change, and the calculation methods vary. Numerous scientometric indexes are available, the most commonly used being the journal impact factor (JIF). Despite its popularity, however, this measure has been heavily criticized [2]. Several alternatives exist, such as the Hirsch index (h-index) [3]. Newer options, such as Eigenfactor score (ES) and scientific journal ranking (SJR), use computational models that measure average prestige per paper, taking into consideration the source of the citations [4].

In addition to the scientific merit of a paper (as measured by citations), the ability to reach a broad audience is another essential measure of scientific impact. With increasing Internet availability, journals are more and more using an open-access (OA) publication model. Thus, journals also act as service providers for information distribution. Confirming this, it has been shown that OA journals receive more citations than subscription-based journals [5,6].

Usage of the OA model for medical journals is similarly increasing. Further to the previously existing submission fees and page charges for medical journals, applying an article processing/publishing charge (APC) with the OA model is an important economic issue for medical professionals and journals. There are currently two major OA models: publishing all papers as OA (all-OA), with or without an APC, and optional OA publishing (hybrid-OA).

Intradisciplinary studies of the various scientometric indexes are required since the number of articles, the citation counts, and citation periods for each discipline are quite different (so interdisciplinary evaluations would be biased). Although several studies comparing the scientometric data of journals according to publication policy are available in other areas of medicine [7], this subject has not been covered in clinical allergy and immunology. Moreover, no study has previously compared the scientometric data of all-OA and hybrid-OA journals in these fields. Therefore, in this study, we aimed to (i) acquire scientometric data for journals publishing in clinical allergy and immunology, and then (ii) investigate this data in terms of the journals’ all-OA and hybrid-OA publication policies.

## Materials and methods

This study included journals listed in the Science Citation Index Expanded (SCIE) that publish in clinical allergy and immunology. To provide homogeneity of analysis, journals that had published fewer than 30 citable documents in the previous three years or fewer than 10 in the last year, multidisciplinary journals that publish fewer than 25% of their original studies in clinical allergy and immunology, and invite-only journals were all excluded from the study. Thus, we included 35 journals in the final analysis.

The scientific and bibliometric data used were obtained from the publicly available data of the Clarivate Analytics InCites and Scimago Journal & Country Rank websites in December 2020. Data for citable documents (five years, 2015-2019), and total citations (five years, 2015-2019), the ratios of articles printed as OA to total citable articles (percentage of citable OA documents) for the 35 journals, together with their ES and JIF, were obtained from Web of Science InCites [8]. SJR and h-index were accessed from the Scimago Journal & Country Rank database [9]. The quartiles for the journals according to scientometric data were recalculated using the SPSS Statistics software package.

Additionally, journal-related data were accessed from the websites of the journals and publishers. The journals’ manuscript types (reviews, original articles, case presentations, and others) were categorized by examining the individual journal websites. The journals were consequently categorized into two: (i) journals that publish all papers, and (ii) journals that publish all articles except case presentations. The journals’ OA publication policies were categorized as (i) all-OA with APC and (ii) hybrid-OA. The fees requested from the authors, timings, amounts and intended purpose of the payments were collected from the journal websites and publishers’ publicly available data. The US dollar (USD) was used as a common currency to measure the fees.

SPSS for Windows version 23.0 (IBM Corp., Armonk, NY) was used for data analysis. Continuous variables were presented as median (minimum-maximum), and categorical data were expressed as values and percentages. Chi-square tests were used for categorical data, Spearman’s rho correlation was used for categorical correlations, and the Mann-Whitney U test was used for continuous variables. A statistical significance limit of p < 0.05 was accepted for all statistical tests.

## Results

Thirty-five journals in the SCIE that publish in clinical allergy and immunology were evaluated in this study. The bibliographic and scientometric data and publication policies are summarized in Table 1. Nearly two-thirds of these journals published all types of papers. Although 15 journals published all articles as OA, the median ratio of OA to non-OA documents in all journals was 6.97%. Seventeen journals charged a mandatory fee for publication. Two journals (one journal with no cost for OA publishing, the other all-OA with APC) required 30 and 40 USD submission fees, respectively, before manuscript acceptance. Two journals with a hybrid-OA publication policy had page charges of 80 and 360 USD after acceptance. The median OA APC fee was 3000 USD (Table 1).

**Table 1:** Bibliographic and scientometric data and publication policies of clinical allergy and immunology journals Data were presented as frequency (percent) for categorical and median (minimum-maximum) for continuous variables. OA: Open-access, JIF: Journal impact factor, SJR: Scientific Journal Ranking score, ES: Eigenfactor score, all-OA with APC: publishing all papers as OA with article processing/publishing charge, hybrid-OA: programs for optional OA publishing, all-OA without APC: publishing all papers as OA without a cost for OA publishing, APC: article processing/publishing charge, USD: US dollar.

Types of published papers			
	All types of papers		22 (62.9%)
	No case reports		13 (37.1%)
Bibliographic information			
	Citable documents	2015	93 (30-311)
		2016	98 (31-314)
		2017	79 (26-304)
		2018	92 (27-534)
		2019	92 (27-296)
		5-year total	456 (150-1759)
	Total citations	2015	1553 (132-41392)
		2016	1906 (171-46218)
		2017	2586 (179-49229)
		2018	2926 (214-51978)
		2019	3219 (242-52714)
		5-year total	12606 (938-241234)
	% of citable OA documents		6.97 (0-100)
Scientometric indexes			
	h-index		53 (16-279)
	JIF		3.5 (0.78-10.22)
	SJR		1.03 (0.236-3.7)
	ES		0.003 (0.0004-0.0774)
Publication policy			
	All OA		15 (42.9%)
	Hybrid OA		20 (57.1%)
Publication fee			
	Mandatory		17 (48.5%)
	Optional		18 (51.5%)
Fee timing			
	Before		1 (2.85%)
	After		33 (94.3%)
	Both		1 (2.85%)
Open access participation			
	All OA with APC		14 (40%)
	Hybrid OA		20 (57.1%)
	Free OA		1 (2.9%)
APC (USD) (n = 34)			3000 (185-4800)

We evaluated four different scientometric indexes. Quartile distributions of journal rankings by these four indexes were compared using Spearman’s correlation coefficient (Table 2). Very strong positive correlations were observed between SJR and JIF and between ES and h-index. ES had strong positive correlations with SJR and JIF. There were moderate correlations of h-index with SJR and with JIF. The top 10 journals ranked by SJR and JIF were similar, while the other scientometric indexes produced different rankings (Table 3).

**Table 2:** Spearman’s rho correlation coefficients of scientometric indexes of clinical allergy and immunology journals JIF: Journal impact factor, SJR: Scientific Journal Ranking score, ES: Eigenfactor score.

	SJR	h-index	JIF
h-index	0.387	-	-
JIF	0.742	0.445	-
ES	0.502	0.808	0.510

**Table 3:** Side by side comparison of top 10 clinical allergy and immunology journals in different scientometric indexes SJR: Scientific Journal Ranking score, JIF: Journal impact factor, ES: Eigenfactor score.

SJR (reference)	JIF	ES	h-index
#1	#1	#1	#1
#2	#2	#2	#2
#3	#3	#3	#4 (↑1)
#4	#5 (↑1)	#15 (↑11)	#10 (↑6)
#5	#22 (↑17)	#10 (↑5)	#13 (↑8)
#6	#6	#19 (↑13)	#19 (↑13)
#7	#19 (↑12)	#4 (↓3)	#20 (↑13)
#8	#14(↑6)	#13 (↑5)	#5 (↓4)
#9	#8 (↓1)	#5 (↓4)	#24 (↑15)
#10	#4 (↓6)	#11 (↑1)	#27 (↑17)

When the percentages of citable OA documents were compared with scientometric and bibliometric data, a moderate negative correlation with ES (R = −0.493, p = 0.003, Spearman's rho correlation) and strong negative correlation with h-index (R = −0.543, p = 0.001, Spearman's rho correlation) were observed. There was no correlation of percentages of citable OA documents with JIF and SJR. The citable OA document percentage had the strongest negative correlation with APC cost (R = −0.661, p = 0.0001, Spearman’s rho correlation). The citable OA document percentage was also negatively correlated with citable documents (five years) (R = −0.479, p = 0.005, Spearman's rho correlation), and total citations (five years) (R = −0.514, p = 0.002, Spearman's rho correlation).

The bibliographic and scientometric data for clinical allergy and immunology journals that publish as all-OA with APC and hybrid-OA were also compared. Data regarding one journal that applies an OA policy with no fees charged were not included in the comparison. The median percentage of citable OA documents in hybrid OA journals was 3.26%. Also, in these journals, h-index and ES were significantly higher (p = 0.001, Mann-Whitney U test), while SJR and JIF were similar. Interestingly, we observed that hybrid-OA journals published 2.17 times more documents than all-OA with APC journals in five years (p = 0.001, Mann-Whitney U test); however, the five-year citation counts of hybrid-OA journals were 6.17 times more than all-OA with APC journals (p = 0.001, Mann-Whitney U test). Notably, all journals within the first quartiles for h-index and ES were hybrid-OA journals. When APC fees were compared, it was found that all-OA journal fees were 2895 USD lower than the median APC charge for hybrid-OA publishing journals (p = 0.0001, Mann-Whitney U test) (Table 4).

**Table 4:** Comparison of bibliographic and scientometric data of clinical allergy and immunology journals with all OA with APC and hybrid OA publishing policies Data were presented as frequency (percent) for categorical and median (minimum-maximum) for continuous variables. OA: open-access, all-OA with APC: publishing all papers as OA with article processing/publishing charge, hybrid-OA: programs for optional OA publishing, JIF: Journal impact factor, SJR: Scientific Journal Ranking score, ES: Eigenfactor score, USD: US dollar. *p < 0.05, Mann-Whitney-U test.

			Hybrid-OA (n = 20)	All-OA with APC (n = 14)
Types of published papers				
	All types of papers		13 (65%)	8 (57.1%)
	No case reports		7 (35%)	6 (42.9%)
Bibliographic information				
	Citable documents	2015*	116 (35-311)	66 (30-309)
		2016*	112 (35-314)	59 (31-221)
		2017*	118 (42-304)	57 (26-207)
		2018*	132 (36-534)	66 (27-323)
		2019*	119 (27-296)	71 (30-241)
		5 year total*	697 (181-1759)	320 (150-1301)
	Total citations	2015*	3810 (132-41392)	563 (173-1751)
		2016*	4107 (171-46218)	689 (224-2073)
		2017*	4491 (179-49229)	673 (260-2836)
		2018*	4576 (214-51978)	752 (300-3743)
		2019*	5038 (242-52417)	815 (380-5081)
		5-year total*	21844 (938-241234)	3538 (1359-14317)
	% of citable OA documents*		3.26 (0-13.3)	100
Scientometric indexes				
	h-index*		89 (21-279)	28 (16-73)
	H-index (first quartile)		9 (45%)	0 (0%)
	JIF		3.66 (0.78-10.22)	2.91 (1.06-5.17)
	JIF (first quartile)		7 (35%)	2 (14%)
	SJR		1.12 (0.23-3.7)	0.83 (0.34-1.37)
	SJR (first quartile)		8 (40%)	2 (14%)
	ES*		0.005 (0.0004-0.0774)	0.0019 (0.0004-0.0153)
	ES (first quartile)		9 (45%)	0 (0%)
Publication fee				
	For submission		Free	40 (1 journal)
	For page charges		80-360 (2 journals)	Free
	APC* (USD)		3570 (2850-4800)	675 (185-3060)

## Discussion

Scientometric indexes are widely used as decision-making tools in the medical field, although none are ideal. Most scientometric indexes have been heavily criticized. First and foremost, JIF has been criticized, for being discipline-specific and biased towards journals that publish reviews, among other reasons [10,11]. Consistent with the criticisms, three out of four articles published in top-tier medical journals have lower citation rates than the JIF of the journal [2]. The h-index has been criticized because it creates a positive bias toward more senior researchers and longer-running journals. Other alternatives include the ES and SJR scores, which consider citation count and citation prestige but have only recently been used and are yet to be compared with other indexes in each discipline [3,12]. One of the aims of this study was to demonstrate and compare the scientometric data of clinical allergy and immunology journals.

This study has identified very strong positive correlations between SJR and JIF, and ES and h-index. To our knowledge, it is the first study to compare the scientometric data of clinical allergy and immunology journals and show such relationships. In a similar study comparing scientometric indexes, for journals on radiology, nuclear medicine, and medical imaging, among others, the R correlation coefficients between scientometric indexes were much higher than those obtained here [13]. In a study of sleep science journals, the authors observed that the journal with the highest JIF was a review journal (it had a JIF nearly double that of its closest competitor). However, according to the h-index, both journals were ranked in the first place [3]. There are similar correlations to those found here in a study on hematology journals [14].

Our analysis also demonstrated that the top 10 journal lists for SJR and JIF were similar, while the other indexes produced diverse rankings. Similar results were published in anatomy and morphology; there were no correlations among JIF, ES, and SJR, where the first journal according to JIF was ranked twentieth according to ES and third according to SJR [4]. In our study on hematology journals, these ranking comparisons were similar as well [14]. These results, along with the current data, indicate the discipline and scientometric index specificity of the comparisons.

Our results indicate that in clinical allergy and immunology, the relationships of SJR to JIF and ES to h-index can be used interchangeably to evaluate journals. Previously, Rizkallah and Sin recommended using JIF and other metrics, such as ES, which considers the quantity and the quality of the citations [15]. Roldan-Valadez et al. recommended using SJR and ES for journals with a JIF of ≥1 [1]. It should also be noted that The San Francisco Declaration on Research Assessment (DORA) recommends against the use of journal-based metrics and also advises that the emphasis on JIF as a promotional tool be significantly reduced [16]. In light of these results, the use of SJR and ES together could be a logical option in clinical allergy and immunology. As these results are discipline-specific, studies for other subject areas are warranted.

Disseminating scientific research to a broader audience as widely and quickly as possible has become crucial to the fundamental goal of achieving scientific impact. The OA publication model has been employed as a result, including in medical journals. This shift caused a change in researcher behavior regarding how authors evaluate journals to determine the required funding for their scientific studies. Authors started to deem OA a vital factor in journal selection when submitting research [17], and, over time, support for and financial contributions to OA publications have increased dramatically [6]. Some data support the idea that the OA publication model results in more citations than the subscription-only one. A paper-based study showed that OA journals receive 1.3 times more citations than their subscription-only counterparts [18]. In psychiatric journals, OA publishing was one of the main factors that increased citation count [5]. However, OA did not affect citations in the field of ophthalmology [19].

In this study, we have shown that most journals in clinical allergy and immunology use the hybrid-OA publication model, but the median of citable OA documents in these journals is extremely low (3.26%)-lower, in fact, than in our previous study on hematology journals (5.94%) [14]. Fifteen journals used the all-OA model, and 14 charged an APC. In our hematology journal study, we could not demonstrate a significant difference in scientometric indexes in favor of the all-OA publication model [14]. In this study, we have presented an adverse effect of the all-OA publication model on h-index and ES. Notably, all journals within the first quartiles for h-index and ES were hybrid-OA journals. These results conflict with the literature finding that the OA publication model causes an increase in citation counts. These results may be discipline-specific and should be further evaluated in a paper-specific manner.

The economic aspect of publication in clinical allergy and immunology is also an important point to consider since the median APC charge for OA publishing is 3000 USD. It was previously demonstrated that the number of OA journals and APCs increases every year [6]. In our hematology journal study, we found that all-OA journals were median 900 USD cheaper than hybrid-OA hematology journals, with similar scientometric indexes [14]. Although the APC gap is much higher in clinical allergy and immunology journals (where the median is 2895 USD lower for all-OA journals), a submitting researcher should also note the significant gap between the scientometric indexes for all-OA and hybrid-OA journals. Clearly, it is important to evaluate several scientometric indexes along with the economic variables. Further studies should focus on the difference in scientometric data between papers published as OA or subscription-only in hybrid-OA journals.

Our study had certain limitations. It was planned with available data on a journal rather than a paper basis. Individual paper-based studies on OA may show different results. Other significant limitations were the bibliographic, scientometric data and that APCs were obtained from databases. APCs may be subject to waivers or discounts, and journals may choose to publish complimentary OA themselves as well.

## Conclusions

When evaluating scientometrics in any science field, one should know the advantages and disadvantages of the scientometric data and the extent to which they correlate with one another. We recommend the use of SJR and ES together as a logical option in clinical allergy and immunology. Although there is a huge APC gap between all-OA and hybrid-OA journals, all journals within the first quartiles for h-index and ES were hybrid-OA. Our results conflict with the literature regarding previous findings that the OA publication model causes an increase in citation counts. Paper-based studies should further confirm these results.
